# Cardiac ISL1-Interacting Protein, a Cardioprotective Factor, Inhibits the Transition From Cardiac Hypertrophy to Heart Failure

**DOI:** 10.3389/fcvm.2022.857049

**Published:** 2022-03-17

**Authors:** Youchen Yan, Tianxin Long, Qiao Su, Yi Wang, Ken Chen, Tiqun Yang, Guangyin Zhao, Qing Ma, Xiaoyun Hu, Chen Liu, Xinxue Liao, Wang Min, Shujuan Li, Dihua Zhang, Yuedong Yang, William T. Pu, Yugang Dong, Da-Zhi Wang, Yili Chen, Zhan-Peng Huang

**Affiliations:** ^1^Department of Cardiology, Center for Translational Medicine, Institute of Precision Medicine, The First Affiliated Hospital, Sun Yat-sen University, Guangzhou, China; ^2^NHC Key Laboratory of Assisted Circulation, Sun Yat-sen University, Guangzhou, China; ^3^Laboratory Animal Center, The First Affiliated Hospital, Sun Yat-sen University, Guangzhou, China; ^4^Department of Cardiology, Boston Children’s Hospital, Harvard Medical School, Boston, MA, United States; ^5^School of Data and Computer Science, Sun Yat-sen University, Guangzhou, China; ^6^Key Laboratory of Machine Intelligence and Advanced Computing, Ministry of Education, Sun Yat-sen University, Guangzhou, China; ^7^Department of Nephrology, The First Affiliated Hospital, Sun Yat-sen University, Guangzhou, China; ^8^National-Guangdong Joint Engineering Laboratory for Diagnosis and Treatment of Vascular Diseases, Guangzhou, China

**Keywords:** heart failure, cardiac hypertrophy, CIP, gene regulation, cardiac remodeling

## Abstract

Heart failure is characterized by the inability of the heart to pump effectively and generate proper blood circulation to meet the body’s needs; it is a devastating condition that affects more than 100 million people globally. In spite of this, little is known about the mechanisms regulating the transition from cardiac hypertrophy to heart failure. Previously, we identified a cardiomyocyte-enriched gene, CIP, which regulates cardiac homeostasis under pathological stimulation. Here, we show that the cardiac transcriptional factor GATA4 binds the promotor of CIP gene and regulates its expression. We further determined that both CIP mRNA and protein decrease in diseased human hearts. In a mouse model, induced cardiac-specific overexpression of CIP after the establishment of cardiac hypertrophy protects the heart by inhibiting disease progression toward heart failure. Transcriptome analyses revealed that the IGF, mTORC2 and TGFβ signaling pathways mediate the inhibitory function of CIP on pathologic cardiac remodeling. Our study demonstrates GATA4 as an upstream regulator of CIP gene expression in cardiomyocytes, as well as the clinical significance of CIP expression in human heart disease. More importantly, our investigation suggests CIP is a key regulator of the transition from cardiac hypertrophy to heart failure. The ability of CIP to intervene in the onset of heart failure suggests a novel therapeutic avenue of investigation for the prevention of heart disease progression.

## Introduction

Heart failure (HF), characterized by insufficient cardiac output to meet the body’s needs, is generally believed to be a clinical consequence of cardiac remodeling. Pressure overload of the left ventricle induced by various clinical conditions, such as hypertension and aortic stenosis, triggers pathological cardiac remodeling (1). Indeed, clinical data revealed that hypertension is a major risk factor for the development of HF (2). Pathological changes of cardiac cells, including hypertrophic growth of cardiomyocytes, necrosis and apoptosis of cardiomyocytes, and activation of cardiac fibroblasts, are closely linked to cardiac remodeling (3, 4). A complex gene regulatory network is thought to control these cellular processes (5). Since the recognition that cardiac remodeling is a key process leading to the development of HF, the slowing or reversing of remodeling has become a goal of HF therapy (1). Therefore, fully understanding the underlying molecular mechanism of cardiac remodeling is a prerequisite for developing new HF therapies. Although several important signaling pathways, such as IGF (6, 7), TGF-beta (8, 9), Mitogen-activated protein kinases (10, 11), Calmodulin-Calcineurin signaling (12, 13), have been identified and well-studied in cardiac hypertrophy, a comprehensive understanding of the molecular mechanisms responsible for progression toward HF remains elusive.

Recently, we identified a striated muscle-enriched protein, CIP (14). CIP is predominately expressed in cardiomyocytes in the heart. Loss-of-function of CIP promotes pressure overload-induced cardiac remodeling and leads to premature HF *in vivo* through modulation of the FOXO1 signaling pathway (15). Human genetic studies revealed that mutation of the CIP gene is associated with human dilated cardiomyopathy (16). Interestingly, our recent study demonstrated the expression of CIP in skeletal muscle plays an important role in regulating nucleus positioning in multinucleated muscle fibers (17). Although overexpression of CIP inhibits hypertrophic growth of cardiomyocytes (14), whether CIP is able to inhibit or even reverse cardiac remodeling after disease status has been established remains unanswered. In this study, we show how CIP expression is regulated in cardiomyocytes and diseased human hearts. Furthermore, we demonstrate that CIP inhibits cardiac remodeling and protects the heart from HF after cardiac hypertrophy has been established, providing evidence of a role for CIP in cardiac hypertrophy and HF treatment.

## Results

### Cardiac Transcription Factor GATA4 Binds to the Promotor of Cardiac ISL1-Interacting Protein and Regulates Its Expression

**FIGURE 1:**
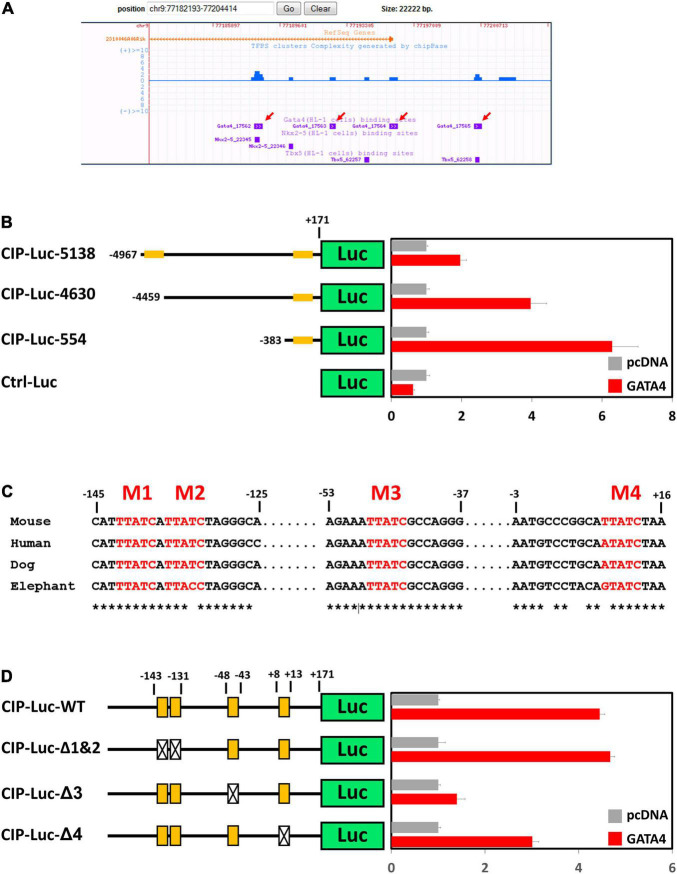
GATA4 regulates the expression of cardiac ISL1-interacting protein (CIP). **(A)** Distribution of reported binding sequences for cardiac transcriptional factors around the transcriptional starting site of CIP gene in a genome browser. Red arrows indicate GATA4 binding sequences. **(B)** Luciferase reporter assay of reporters with full length or truncated CIP promotors with or without GATA4 activation. Orange bars indicate potential GATA4 binding sites. **(C)** Conservation of potential GATA4 binding motifs in mammalian, which are shown in red letters. Asterisks indicate the conserved nucleotides. **(D)** Luciferase reporter assay of reporters with full wildtype or mutant CIP promotors with or without GATA4 activation. Orange boxes indicate potential GATA4 binding motifs. Boxes with cross indicate mutant motifs.

Cardiac ISL1-interacting Protein (CIP) (14) or Muscle-enriched A-type Lamin-Interacting Protein (MLIP) (18) has been previously identified as a striated muscle-enriched gene. In the heart, CIP is dominantly expressed in cardiomyocyte cells. However, transcriptional factor(s) that control the expression of CIP in cardiomyocytes have not yet been investigated. Genome-wide binding sites for multiple cardiac transcriptional factors, including GATA4, Tbx5, Nkx2-5, Mef2A, and SRF, have been carefully investigated (19). Binding sites for GATA4, Tbx5, and Nkx2-5 were detected in the promotor region of CIP (Figure 1A). Knock-down of GATA4 alone or both GATA4 and mef2a decreased the expression of CIP in cardiac cells (Supplementary Figure 1), indicating GATA4 is a key transcriptional factor for the expression of CIP. To determine whether CIP is directly regulated by GATA4, we generated luciferase reporters controlled by a series of CIP promotor sequence variants, each of which contains the GATA4-binding sequences detected by previous CHIP-seq assay (19). In transient cell-based luciferase assays, all CIP-Luc reporter variants were responsive to GATA4 transactivation. The 554bp CIP-Luc reporter construct, containing a proximal GATA4 binding sequence, was the most responsive one in the assay, indicating a functional GATA4 enhancer located in this binding sequence (Figure 1B). Four putative GATA4 binding motifs, which are evolutionarily conserved, were found in 554 bp CIP promotor fragment (Figure 1C). To determine which of the binding motifs were functional, we generated three mutant variants of the 554 bp CIP-Luc reporter that contained mutant versions of motifs 1 and 2, motif 3, and motif 4, respectively. The results of the luciferase assay revealed that mutations in motif 3 and motif 4, but not motif 1 and 2, decreased the responsivity of 554 bp CIP-Luc reporter to GATA4 transactivation (Figure 1D), suggesting motif 3 and motif 4 are functional GATA4 binding motifs in CIP promotor. Therefore, our data demonstrated that GATA4 is a key transcriptional factor regulating the expression of CIP in cardiomyocytes.

### Decreased Expression of Cardiac ISL1-Interacting Protein Correlated With Dysregulated Oxidative Phosphorylation Pathway Activity in Diseased Human Hearts

**FIGURE 2:**
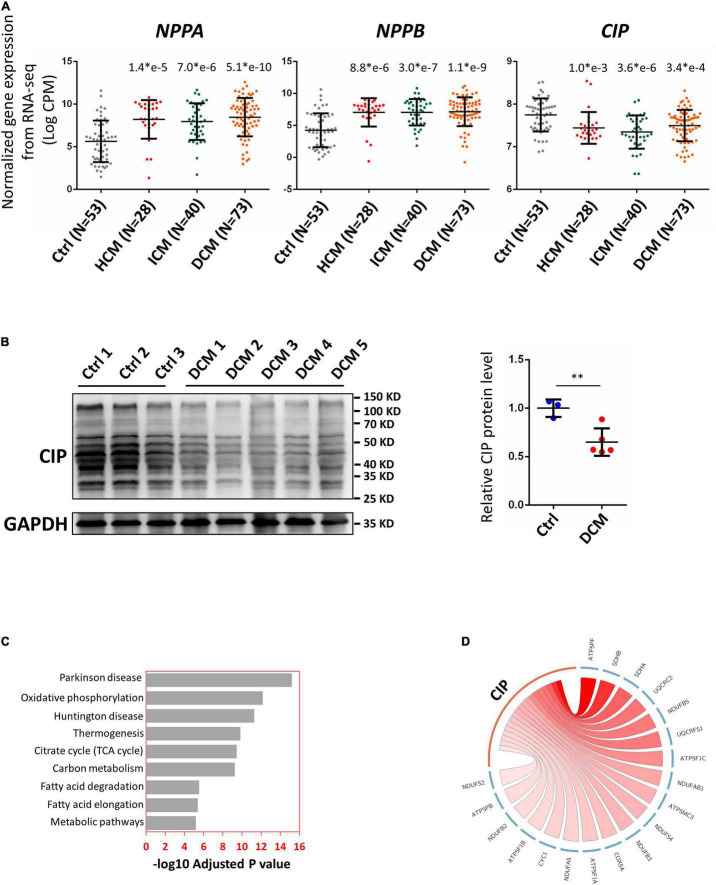
Cardiac ISL1-interacting protein expression is down-regulated in diseased human hearts. **(A)** Relative gene expression indicated by log counts per million (CPM) from disease human heart RNA-seq data. *N* number for each group is show. The significance between each category of heart disease and control was tested with 1-way ANOVA with *post hoc* Tukey’s test and shown. **(B)** Western blotting detecting the expression of CIP in human hearts with dilated cardiomyopathy (DCM) and controls. GAPDH served as loading control. The significance between groups was tested with 1-way ANOVA with *post hoc* Tukey’s test. ^**^P < 0.01. **(C)** The ranking of enriched KEGG pathways in the gene set having strong expression correlation coefficient with CIP, which is determined by Spearman correlation coefficient (SCC). SCC between CIP and individual genes genome-wide from 194 human hearts was calculated. Pathways were ranked by the adjusted *P* value. **(D)** The expression of CIP has strong expression correlation coefficient with oxidative phosphorylation (OXPHOS) related genes in human heart (*p* = 6.77 × e10^– 13^). OXPHOS genes with SCC > 0.4 are shown in Circos plot. The color of line linked between CIP and each gene indicates the expression correlation coefficient. A darker line suggests a stronger expression correlation coefficient.

In order to further investigate the relevance of CIP in human cardiac diseases, we collected a total of 194 RNA-seq datasets based on analyses of human heart tissue from the NCBI public database, including 53 non-failing heart samples (NF), 28 hypertrophic cardiomyopathy samples (HCM), 40 ischemic cardiomyopathy samples (ICM), and 73 dilated cardiomyopathy samples (DCM). After normalizing the data from different batches, we found that expression of CIP was significantly decreased in all groups of diseased hearts, while cardiac disease markers, NPPA and NPPB, were significantly upregulated (Figure 2A). Furthermore, the downregulation of CIP in human DCM hearts was further confirmed by Western blotting analysis (Figure 2B). Next, we examined the data to detect genes co-expressed with CIP to define a potential CIP-based gene regulation network. Human genes were divided into multiple modules based on the Spearman correlation coefficient score of genes, then the module containing CIP was further subjected to KEGG pathway analysis. Pathways related to “Parkinson disease,” “oxidative phosphorylation,” “Huntington disease,” and “metabolic pathways” were on the top of the list (Figure 2C). Given that we focused on cardiac disease in this study, our data suggest that CIP was involved in the regulation of OXPHOS pathway. OXPHOS-related genes with the strongest correlation coefficient, including ATP5PF, SDHB, SDHA, etc., are illustrated in a Circos plot (Figure 2D). Together, our data from this genome-wide analyses of human diseased hearts suggest CIP plays an important role in human heart disease by regulating oxidative phosphorylation in cardiomyocyte.

### Cardiac Overexpression of Cardiac ISL1-Interacting Protein Protects the Heart From Disease Progression to Heart Failure

**FIGURE 3:**
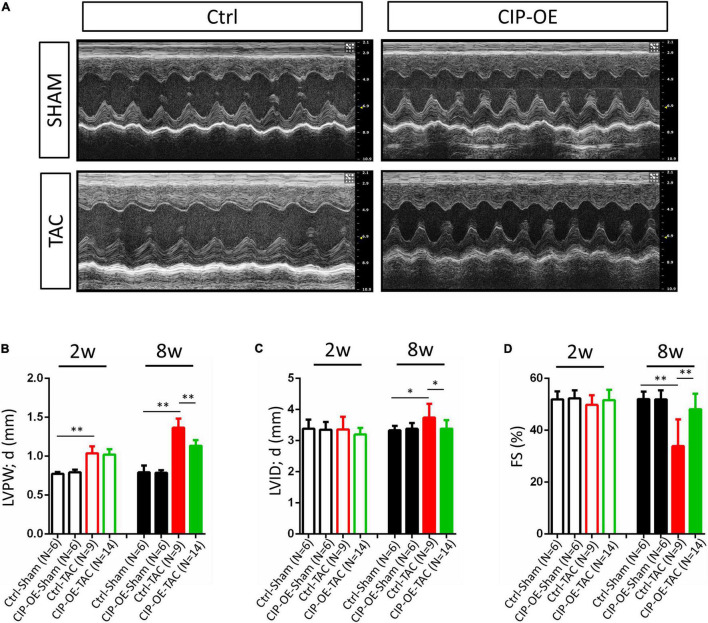
Cardiac-overexpression of CIP preserves cardiac function during the disease progression toward heart failure. **(A)** Representative echocardiographic images from indicated group at 8 weeks after operation. **(B)** Left ventricular posterior wall thickness at end-diastole (LVPW;d), **(C)** Left ventricular internal dimension at end-diastole (LVID;d) and **(D)** Fractional shortening (FS) of CIP-OE mice and their control littermates with TAC or Sham operation determined by echocardiography at 2 weeks (2w) and 8 weeks (8w) after operation. *N* number for each group is show. The significance between groups was tested with 1-way ANOVA with *post hoc* Tukey’s test. *P < 0.05; ^**^P < 0.01.

In a previous study, we reported that overexpression of CIP in the heart before cardiac stress inhibited the stress-induced cardiac mal-remodeling (15). In order to test the therapeutic potential of CIP in interfering the progression of adverse cardiac remodeling, we performed transverse aortic constriction (TAC) surgery, which induced ventricular pressure overload, on an inducible cardiac-specific CIP overexpressing mouse model (Rosa26-CIP-flox; Myh6-MerCreMer, and CIP-OE mice). Mice were administrated Tamoxifen to induce the cardiac-specific overexpression of CIP 2 weeks after TAC surgery (Supplementary Figure 2). The establishment of cardiac remodeling was confirmed at 2 weeks after TAC as indicated by the echocardiography examination of the left ventricular posterior wall (LVPW; Figures 3A,B and Table 1). Cardiac parameters were measured at 2 and 8 weeks post-surgery by echocardiography (Table 1). Compared to the sham-operated group, the ventricular wall became thicker and the ventricular chamber was dilated in control mice when the stress was prolonged (Figures 3A,C). As expected, their cardiac function reflected by fraction shortening (FS) decreased significantly, indicating disease progression to heart failure (Figures 3A,D). In contrast, overexpression of CIP in the heart during stress significantly repressed the thickening of the ventricular wall and chamber dilation. More importantly, cardiac function was preserved by CIP overexpression during the prolonged stress (Figures 3A–D).

**TABLE 1:** Echocardiography examination of cardiac ISL1-interacting protein (CIP)-OE mice and their control littermates with transverse aortic constriction (TAC) or sham operation at different time points after surgery.

	Ctrl; Sham (*N* = 6)		CIP-OE; Sham (*N* = 6)		Ctrl; TAC (*N* = 9)		CIP-OE; TAC (*N* = 14)	
	2 weeks	8 weeks	2 weeks	8 weeks	2 weeks	8 weeks	2 weeks	8 weeks
IVS;d (mm)	0.770 ± 0.065	0.811 ± 0.075	0.784 ± 0.042	0.807 ± 0.057	1.014 ± 0.090**	1.305 ± 0.031**	1.063 ± 0.089	1.188 ± 0.148^#^
IVS;s (mm)	1.421 ± 0.102	1.499 ± 0.096	1.448 ± 0.135	1.494 ± 0.174	1.647 ± 0.136**	1.742 ± 0.158**	1.679 ± 0.130	1.825 ± 0.140
LVID;d (mm)	3.387 ± 0.284	3.332 ± 0.144	3.350 ± 0.250	3.383 ± 0.182	3.361 ± 0.407	3.740 ± 0.441*	3.198 ± 0.214	3.386 ± 0.268^#^
LVID;s (mm)	1.632 ± 0.213	1.600 ± 0.129	1.595 ± 0.115	1.623 ± 0.074	1.696 ± 0.296	2.509 ± 0.706**	1.550 ± 0.197	1.766 ± 0.306^##^
LVPW;d (mm)	0.775 ± 0.021	0.793 ± 0.088	0.793 ± 0.032	0.788 ± 0.033	1.036 ± 0.092**	1.366 ± 0.118**	1.021 ± 0.070	1.133 ± 0.072^##^
LVPW;s (mm)	1.513 ± 0.130	1.494 ± 0.210	1.503 ± 0.071	1.609 ± 0.182	1.739 ± 0.164*	1.803 ± 0.272*	1.734 ± 0.191	1.829 ± 0.181
EF (%)	83.94 ± 2.75	84.07 ± 2.52	84.30 ± 2.68	83.91 ± 2.91	81.99 ± 3.69	62.21 ± 16.10**	83.76 ± 3.49	80.08 ± 6.47^##^
FS (%)	51.93 ± 3.04	52.02 ± 2.88	52.31 ± 3.12	51.91 ± 3.46	49.79 ± 3.74	33.86 ± 10.38**	51.64 ± 3.92	48.11 ± 5.97^##^
LV Mass (mg)	85.89 ± 13.71	87.95 ± 11.87	86.49 ± 9.48	89.49 ± 11.42	128.89 ± 32.07**	224.51 ± 45.51**	121.01 ± 13.79	155.39 ± 20.19^##^
LV Mass (Corrected, mg)	68.71 ± 10.97	70.36 ± 9.50	69.19 ± 7.59	71.59 ± 9.13	103.11 ± 25.66**	179.61 ± 36.41**	96.81 ± 11.03	124.31 ± 16.15^##^
LV Vol;d (μL)	47.44 ± 9.92	45.29 ± 4.72	46.12 ± 8.31	47.03 ± 5.87	47.09 ± 13.32	60.78 ± 18.05	41.17 ± 6.64	47.41 ± 8.87^#^
LV Vol;s (μL)	7.75 ± 2.79	7.24 ± 1.52	7.17 ± 1.30	7.46 ± 0.85	8.77 ± 3.86	25.30 ± 20.09*	6.79 ± 2.30	9.75 ± 4.48^#^
Heart Rate (BPM)	705 ± 17	687 ± 21	711 ± 48	731 ± 16	663 ± 58	655 ± 84	726 ± 32	698 ± 63

*P_Ctrl;Sham (same timepoint) vs. Ctrl;TAC (same timepoint)_ < 0.05; **P_Ctrl;Sham (same timepoint) vs. Ctrl;TAC (same timepoint)_ < 0.01; ^#^P_Ctrl;TAC (same timepoint) vs. CIP–OE; TAC (same timepoint)_ < 0.05; ^##^P_Ctrl;TAC (same timepoint) vs. CIP–OE; TAC (same timepoint)_ < 0.01.

**FIGURE 4:**
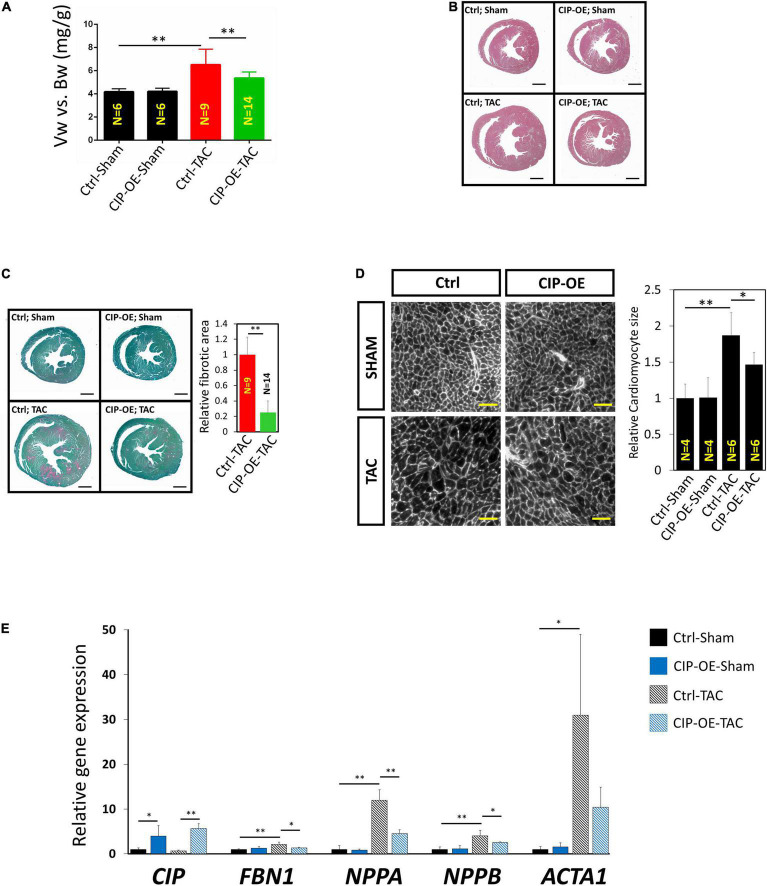
Cardiac ISL1-interacting protein inhibits cardiac remodeling in the transition from cardiac hypertrophy to failure. **(A)** The ratio of ventricle weight vs. body weight of CIP-OE mice and their control littermates at 8 weeks after TAC or sham operation. *N* number of each group is shown. **(B)** Haematoxylin Eosin (H&E) staining of hearts from CIP-OE mice and their control littermates at 8 weeks after TAC or sham operation. Bars = 1 mm. **(C)** Fast green and Sirius red staining of hearts from CIP-OE mice and their control littermates at 8 weeks after TAC or sham operation. The fibrotic area was quantified. Bars = 1 mm. **(D)** Wheat germ agglutinin staining detecting the cross area of cardiomyocytes in TAC- or sham-operated CIP-OE hearts and littermate controls. The size of cardiomyocyte was quantified. Bars = 40 μm. **(E)** qRT-PCR detection of expression of cardiac fibrosis and heart disease marker genes in TAC- or sham-operated CIP-OE hearts and littermate controls. *N* = 4 for each group. The significance between groups was tested with 1-way ANOVA with *post hoc* Tukey’s test. *P < 0.05; ^**^P < 0.01.

Mice were sacrificed at 8 weeks post-surgery for cardiac tissue collection. Consistent with echocardiographic data, significant adverse cardiac remodeling was induced by pressure overload in control mice, which was indicated by the ratio of ventricular weigh vs. body weight (Vw vs. Bw) and histological examination (Figures 4A,B). Overexpression of CIP in the heart resulted in a smaller heart under pressure overload. Consistently, cardiomyocyte size, indicated by its cross area determined by Wheat Germ Agglutinin (WGA) staining, was significantly larger in the control group after TAC but became smaller when CIP was overexpressed (Figure 4C). Heart failure is often accompanied with increased cardiac fibrosis. Sirius red/Fast green staining showed less cardiac fibrosis in CIP-OE hearts under cardiac stress compared to the control group (Figure 4D). We further examined the molecular markers for cardiac disease and fibrosis, including NPPA, NPPB, ACTA1, and FBN1. The induction of these marker gene expression by cardiac stress was significantly repressed by the CIP overexpression (Figure 4E). Together, all these data demonstrated that cardiac overexpression of CIP after the pathological remodeling has been initiated is able to inhibit disease progression and protect the heart from failure.

### The Protective Function of Cardiac ISL1-Interacting Protein Is Mediated by IGF and mTORC2 Signaling Pathways

**FIGURE 5:**
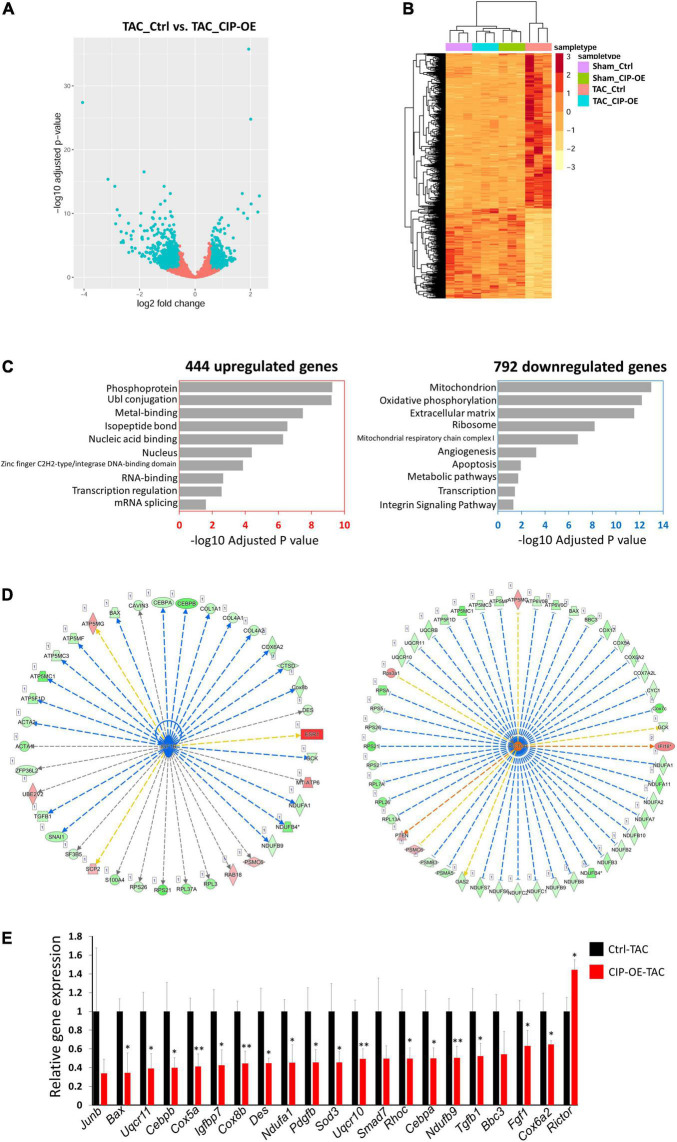
IGF1R and mTORC2 signaling pathways mediates the function of CIP in cardiac protection. **(A)** A volcano plot of all detected genes in RNA-seq. Each dot represent one gene and the blue dots indicate dys-regulated genes in TAC-CIP-OE hearts comparing to TAC-Ctrl hearts. **(B)** A hierarchical clustering heatmap of 1,236 dys-regulated genes in all groups. **(C)** Gene ontology analysis of 444 up-regulated (*p* < 0.05) and 792 down-regulated (*p* < 0.05) genes in TAC-CIP-OE hearts. The GO terms are ranked by the adjusted *P* values. **(D)** Ingenuity Pathway Analysis (IPA) of upstream regulators of dys-regulated genes in CIP-OE heart after 8 weeks of TAC operation. Genes in green indicate their expression in CIP-OE heart is down-regulated. Genes in red indicate their expression in CIP-OE heart is up-regulated. Blue lines indicate that the dysregulation of upstream regulator lead to the down-regulation of the downstream genes, which is consistent with reported data; Red lines indicate that the dysregulation of upstream regulator lead to the up-regulation of the downstream genes, which is consistent with reported data. Yellow lines indicate that the gene regulation is inconsistent with reported data. Gray lines indicate the unknown gene regulation; **(E)** qRT-PCR validation of dys-regulated genes downstream of IGF1R, Rictor or TGF1B. *N* = 3 for each group. The significance between groups was tested with 1-way ANOVA with *post hoc* Tukey’s test. *P < 0.05; ^**^P < 0.01.

In order to investigate the potential mechanism of the protective effect of CIP in cardiac remodeling, we carried out unbiased transcriptome profiling with hearts of CIP-OE mice at 8 weeks post-surgery by performing RNA-seq. In total, 444 genes, including CIP, were significantly up-regulated, while 792 gene were significantly down-regulated in CIP-OE hearts (fold change > 1.5, *p* < 0.05; Figure 5A). A hierarchical clustering heatmap revealed that the dysregulation of these genes was induced by TAC surgery in the control group but rescued by the overexpression of CIP in stressed hearts (Figure 5B). In order to further characterize this subset of genes, 1,236 dysregulated genes were subjected to GO term analysis. Consistent with what we had found in human diseased hearts, genes related to “Mitochondrion” and “Oxidative phosphorylation” were enriched in the down-regulated genes (Figure 5C). To gain more information of the gene regulation network, we further searched for upstream regulators of these dysregulated genes using Ingenuity Pathway Analysis (IPA). Several regulators, including IGF1R (*p* = 7.27e-8; Figure 5D), Rictor (a core component of mTORC2, *p* = 6.25e-14; Figure 5D) and TGFB1 (*p* = 4.76e-7; Supplementary Figure 3), were showed in the top list. It is worth noting that the IGF1R was reported to modulate the activity of FoxO1 through regulating AKT, which is consistent with our previous report that CIP regulated pathological cardiac remodeling through the FoxO1/CnA signaling cascade (15). Dysregulation of downstream targets for IGF1R from IPA analysis, including TGFB1, BAX, CEBRA, CEBRB, COX6A2, COX8B, DES, NDUFA1, and NDUFB9, as well as several downstream targets for Rictor and TGFB1 were further confirmed by qRT-PCR (Figure 5E). All these data suggested that the inhibitory function of CIP on the progression of adverse cardiac remodeling was mediated, at least in part, by IGF and mTORC2 signaling pathways.

## Discussion

Although it has been recognized for a long time that the progression from cardiac hypertrophy to heart failure is detrimental, how this transition process is regulated remains largely unknown. Exploring a new regulator and associated molecular mechanisms connected to this disease progression will promote the development of new therapeutic approaches for heart failure. We have previously identified a striated muscle-enriched expressed gene, CIP, and showed it participated in the modulation of cardiac disease (14, 15). More and more genetic evidences indicate that CIP is a critical gene associated with human muscular diseases, both cardiac and skeletal ones. An exonic mutation of CIP gene was reported to associate with human dilated cardiomyopathy (16). Recently, loss-of-function of CIP induced by DNA mutations was demonstrated to cause myopathy with hyperCKemia in human (20, 21). CIP is specifically expressed in cardiomyocyte in the heart, but the regulatory mechanism of CIP’s expression remains unclear. Here, we reported that GATA4, a key cardiac transcriptional factor, binds to the promotor of CIP and regulates its expression. Interestingly, GATA4 was shown to be upregulated in human failing hearts (22), and its expression decreases in response to the treatment of left ventricular assist device (LVAD) in heart failure (23), indicating other regulatory factors exist for CIP expression control. Significantly, CIP was shown to be down-regulated in various human heart diseases at both the mRNA and protein levels, indicating CIP plays an important regulatory role in the pathogenesis of human heart diseases and could be a target for reversing the adverse effects of the remodeling process and remodeling itself, thereby preventing heart failure.

The molecular mechanism of CIP’s function in protecting the heart was further explored in this study. Several key upstream regulators, including IGF1R, Rictor and TGFB1, were identified. Previously, we showed that CIP regulates the activity of a key cardiac remodeling regulator, Calcineurin, through FoxO1 (15). Here, we found that CIP regulated the activity of IGF1R, an upstream regulator of FoxO1 (24, 25) and played an important role in cardiac hypertrophy (26). Rictor, a core component in mTORC2 (27), displayed increased expression levels in CIP-overexpressing hearts, indicating activation of mTORC2. Indeed, activation of mTORC2 shows a cardioprotective effect in stressed hearts (28). TGFB1, which showed decreased expression in stressed CIP-overexpressing hearts, seems to be another key factor mediating CIP’s function. Although TGFB1 has been widely demonstrated to regulate cardiac fibrosis in heart disease (29, 30), it was also shown to regulate the hypertrophic growth of cardiomyocytes through TAK1 (9). Interestingly, TGFB1 stimulates mitochondrial oxidative phosphorylation in non-cardiomyocytes (31). Consistently, the decreased expression of TGFB1 is coincident with the pattern of down-regulation of “oxidative phosphorylation” genes in the heart when CIP was overexpressed in our study. Indeed, we showed that CIP has a strong expression correlation coefficient with “oxidative phosphorylation” genes in human cardiac samples. In addition, our recent study indicated that CIP modulates oxidative stress by regulating CnA-NFAT-Nox4 signaling cascade in dystrophic cardiomyopathy (32). Further study is required to further dissect how CIP regulates this gene network before it can be pursued as a therapeutic approach for heart failure.

Previously, we demonstrated that CIP functions as a stress sensor and inhibited pathological hypertrophy from base line (15). This observation is a good sign that CIP has the potential for clinical application; however, we often face patients who have already developed severe cardiac sequelae in the hospital. To explore the potential of CIP in this situation, we induced cardiac-specific overexpression of CIP after a disease model of cardiac hypertrophy was established. Our data demonstrated that CIP significantly delayed disease progression and pathological cardiac remodeling, as well as protecting the heart from heart failure during prolonged stress; however, the overexpression could not reverse the hypertrophy. The results suggests that the CIP protein has great therapeutic potential in the treatment of cardiac disease progression toward heart failure. In the future, cardiac CIP overexpression mediated by adeno-associated virus (AAV), which has been well established for transgene expression in the heart and proposed for clinical study (33, 34), should be tested to further support this potential.

## Materials and Methods

### Human Samples

Left ventricular (LV) tissues were collected from patients with end-stage heart failure during heart transplantation performed in the First Affiliated Hospital, Sun Yat-sen University. In brief, diseased hearts were removed at the time of transplantation and LV tissue was subsequently dissected and snap-frozen. We used LV samples from not implanted healthy hearts to serve as controls (Supplementary Table 1). All the procedures followed the protocol approved by the First Affiliated Hospital, Sun Yat-sen University, Guangzhou, China.

### Mice

Cardiac ISL1-interacting Protein-KI-flox mice were generated in a previous study (15). CIP-KI-flox mice, which have a Rosa-CIP allele (the stop codon is present and floxed) were bred with aMHC–Mer–Cre–Mer mice to obtain CIP-OE (CIP-KI-flox; aMHC–Mer–Cre–Mer) mice. Tamoxifen was administrated through intraperitoneal injection to activate the expression of Cre recombinase and the excision of the stop codon for the ectopic expression of CIP transgene in the heart in CIP-OE mice. CIP-KI-flox littermates were used as controls.

### Measurement of Cardiac Function by Echocardiography

Echocardiographic measurements were performed on mice using a Visual Sonics Vevo^®^ 2100 Imaging System (Visual Sonics, Toronto, ON, Canada) with a 40 MHz MicroScan transducer (model MS-550D). Mice were anesthetized with isoflurane (2.5% isoflurane for induction and 0.1% for maintenance). Heart rate and LV dimensions, including diastolic and systolic wall thicknesses, LV end-diastolic and end-systolic chamber dimensions were measured from 2-D short-axis under M-mode tracings at the level of the papillary muscle. LV mass and functional parameters such as percentage of fractional shortening (FS%) and ejection fraction (EF%) were calculated using the above primary measurements and accompanying software.

### Transverse Aortic Constriction Operation

Mice were anesthetized with isoflurane (3–4% isoflurane for induction, 1–2% isoflurane for maintenance). The chest was shaved and cleaned with alcohol. A suture was placed around the front upper incisors and pulled taut so that the neck was slightly extended. The tongue was retracted and held with forceps, and a 20-G catheter was inserted into the trachea. The catheter was then attached to the mouse ventilator via a Y-shaped connector. Ventilation was performed with a tidal volume of 220–240 μl for a 25–30 g mouse and a respiratory rate of 130–140 breaths per min. 100% oxygen was provided to the inflow of the ventilator. The chest was opened through a left 2nd intercostal thoracotomy. The 26-G needle without its sharp tip was put on the ascending aorta. They were tightly ligated together using 7-0 Nylon suture (Ethicon, Edinburgh, Scotland) at the position between brachiocephalic artery and left common carotid artery, and the 26-G needle was removed immediately after ligation. In the sham operation, no ligation was performed. Isoflurane was stopped, and the lungs were slightly overinflated to assist in removal of air in the pleural cavity. Dissected intercostal space and chest skin were closed using 6-0 silk suture (Ethicon, Edinburgh, Scotland). All manipulations were performed by an operator without knowledge of genotype.

### Haematoxylin and Eosin Staining and Fast Green/Sirius Red Collagen Staining

Mouse heart tissues were dissected from the animals, rinsed with PBS and fixed in 4% paraformaldehyde (pH 8.0) overnight. After dehydration through a series of ethanol baths, samples were embedded in paraffin wax according to standard laboratory procedures. Sections of 5 μm were stained with haematoxylin and eosin (H&E) for routine histological examination with light microscope. For Sirius red/fast green collagen staining sections were fixed with pre-warmed Bouins’ solution, 55°C for 1 h then washed in running water. Sections were stained in 0.1% fast green solution for 10 mins then washed with 1% Acetic acid for 2 mins. After rinsing in tape water, sections were stained in 0.1% Sirius resolution for 30 mins. After staining, sections were dehydrated and cleared with Xylene. The images were examined with light scope and quantified with ImageJ software.

### Quantitative RT-PCR and Western Blot Analysis

Total RNAs were isolated using Trizol Reagent (Life Technologies, Carlsbad, CA, United States) from cells and tissue samples. For Quantitative RT-PCR, 2.0 μg RNA samples were reverse-transcribed to cDNA by using random hexamers and MMLV reverse transcriptase (Life Technologies) in 20 μl reaction system. In each analysis, 0.1 μl cDNA pool was used for quantitative PCR. The relative expression of interested genes is normalized to the expression of ACTB or PRKG1. Primers used in this study were listed (Supplementary Table 2). For Western blot analyses, tissue homogenate were cleared by 10,000 × *g* centrifugation for 10 min. Samples were subsequently analyzed by SDS/PAGE and transferred to PVDF membranes that were incubated with 5% non-fat dry milk in TBST and Anti-CIP (1:2,000, 21st Century Biochemical, Marlboro, MA, United States) or Anti-GAPDH (1:5,000, Proteintech, Rosemont, IL, United States) overnight at 4°C and then washed three times with TBST buffer before adding IgG secondary antibody. Specific protein bands were visualized through chemiluminescent detection.

### Constructs, Cell Culture, and Luciferase Reporter Assays

HEK293T cells were cultured in DMEM supplemented with 10% FBS in a 5% CO^2^ atmosphere at 37°C. Wildtype, mutant or truncated CIP promoter sequences were cloned into multiple cloning sites of the pGL3-Basic vector (Promega, Madison, WI, United States) to generate CIP-Luc reporters used in this study. The indicated combinations of CIP-Luc reporter, pRL Renilla reporter (internal control) and GATA4 construct were transfected into HEK293T cells with PEI reagents. Forty Eight hours after transfection, cell extracts were prepared and luciferase activity was determined. For dual-luciferase assay, normalized firefly luciferase expression from triplicate samples in 12-well plates relative to renilla luciferase expression was calculated.

### Mouse Heart RNA-Seq Data Analyses

Total RNAs from mouse heart were used to perform RNA-seq in BGI Genomics (Wuhan, China). RNA-seq reads were mapped to mouse genome mm10 by STAR and reads counts were calculated with FeatureCounts. Expression analysis was run in RStudio. DESeq2 was employed to perform statistical analysis of differential gene expression. An adjusted *P* value of 0.05 were used as cutoff to identify differentially regulated genes. Volcano plot were performed with the ggplot2 library. Hierarchical clustering heatmap was made with the pheatmap library. The raw data of RNA-seq in this study were deposited in Gene Expression Omnibus (GEO) database of the National Center for Biotechnology Information (NCBI) (Accession: GSE194149).

### Human Heart RNA-Seq Data Collection and Analyses

Human diseased heart RNA-seq data, including GSE57344, GSE71613, GSE116250, GSE46224, GSE108157, GSE55296, GSE120836, and GSE130036, were downloaded from NCBI database. Gene-level quantification were calculated by featureCounts-v1.6.3. To perform strand-specific reads counting, the strand type (non-strand, stranded, reversely stranded) of each sample was inferred from sorted bam file using infer_expriment.py (3.0.0). Then we provided featureCounts with strand type information to calculate read counts of every gene in each sample and merged the quantification results together to make an expression matrix for differential gene expression analysis. Differential gene expression analysis was performed using DESeq2-1.24.0. The design matrix in DESeq2 model was written as “∼series + gender + phenotype” to adjust the differences between data series and gender. Only differential expressed genes with FDR < 0.05 and log2FoldChange >0.25 identified by DESeq2 were kept. Then, we applied the classic weighted correlation network analysis (WGCNA) algorithm for co-expression analysis. The R implementation of WGCNA (version: 1.68) was used in our study.

### Statistics

Values are reported as means ± SEM unless indicated otherwise. Statistical significance was determined with ANOVA. For multiple group comparisons, a *post hoc* Tukey’s test was performed when ANOVA reached significance. Values of *P* < 0.05 were considered statistically significant.

## Data Availability Statement

The datasets presented in this study can be found in online repositories. The names of the repository/repositories and accession number(s) can be found below: GEO, GSE194149.

## Ethics Statement

The studies involving human participants and animal study were reviewed and approved by the Medical Ethics Committee of the First Affiliated Hospital, Sun Yat-sen University. The patients/participants provided their written informed consent to participate in this study. Written informed consent was obtained from the owners for the participation of their animals in this study.

## Author Contributions

Z-PH and D-ZW conceived the project, designed and analyzed the experiments, and wrote the manuscript. YoY, TL, TY, and XH performed molecular biology experiments. CL, XL, SL, YC, and YD contributed to the human sample acquisition and western blotting analysis. QS, QM, and GZ generated CIP-OE mice, performed transverse aortic constriction surgery, and collected mouse heart samples. QS and GZ contributed to the echocardiographic data acquisition and analysis. TY, DZ, and XH contributed to the histological and immunofluorescent data acquisition and analysis. YW, KC, and YuY contributed to bioinformatic analyses of RNA-seq data. WM and WP supervised the CIP-KO mice generation and surgery and reviewed the manuscript. All authors contributed to the article and approved the submitted version.

## Conflict of Interest

The authors declare that the research was conducted in the absence of any commercial or financial relationships that could be construed as a potential conflict of interest.

## Publisher’s Note

All claims expressed in this article are solely those of the authors and do not necessarily represent those of their affiliated organizations, or those of the publisher, the editors and the reviewers. Any product that may be evaluated in this article, or claim that may be made by its manufacturer, is not guaranteed or endorsed by the publisher.
