# Supplementary material for: Cardiac ISL1-Interacting Protein, a Cardioprotective Factor, Inhibits the Transition From Cardiac Hypertrophy to Heart Failure
Source: Front Cardiovasc Med. 2022 Mar 17;9:857049. doi: 10.3389/fcvm.2022.857049 (PMC8970336; doi:10.3389/fcvm.2022.857049)
Supplement: Supplementary file 1 [file Data_Sheet_1.pdf]

Supplemental Figure 1

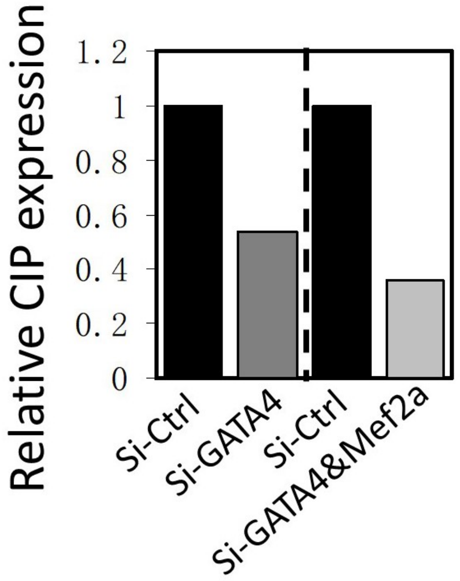

Supplemental Figure 1. The transcriptional factor GATA4 regulates the expression of CIP. The expression data of CIP when GATA4 and/or Mef2a were knocked down in HL1 cells from public database (GSE21529).

Supplemental Figure 2

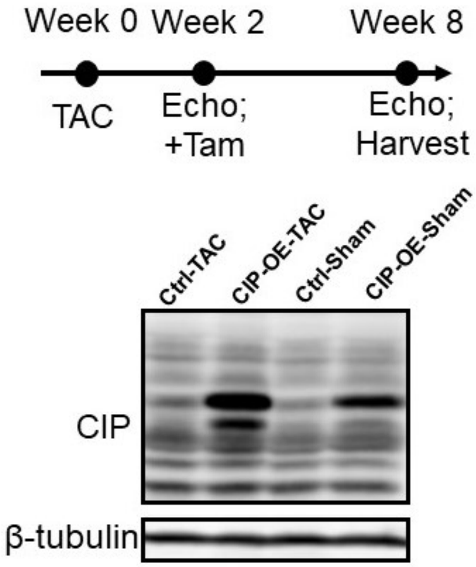

Supplemental Figure 2. Design of cardiac stress experiment and detection of CIP overexpression in mouse heart. Cardiac overexpression of CIP protein was detected by western blot. β–tubulin served as internal control.

Supplemental Figure 3

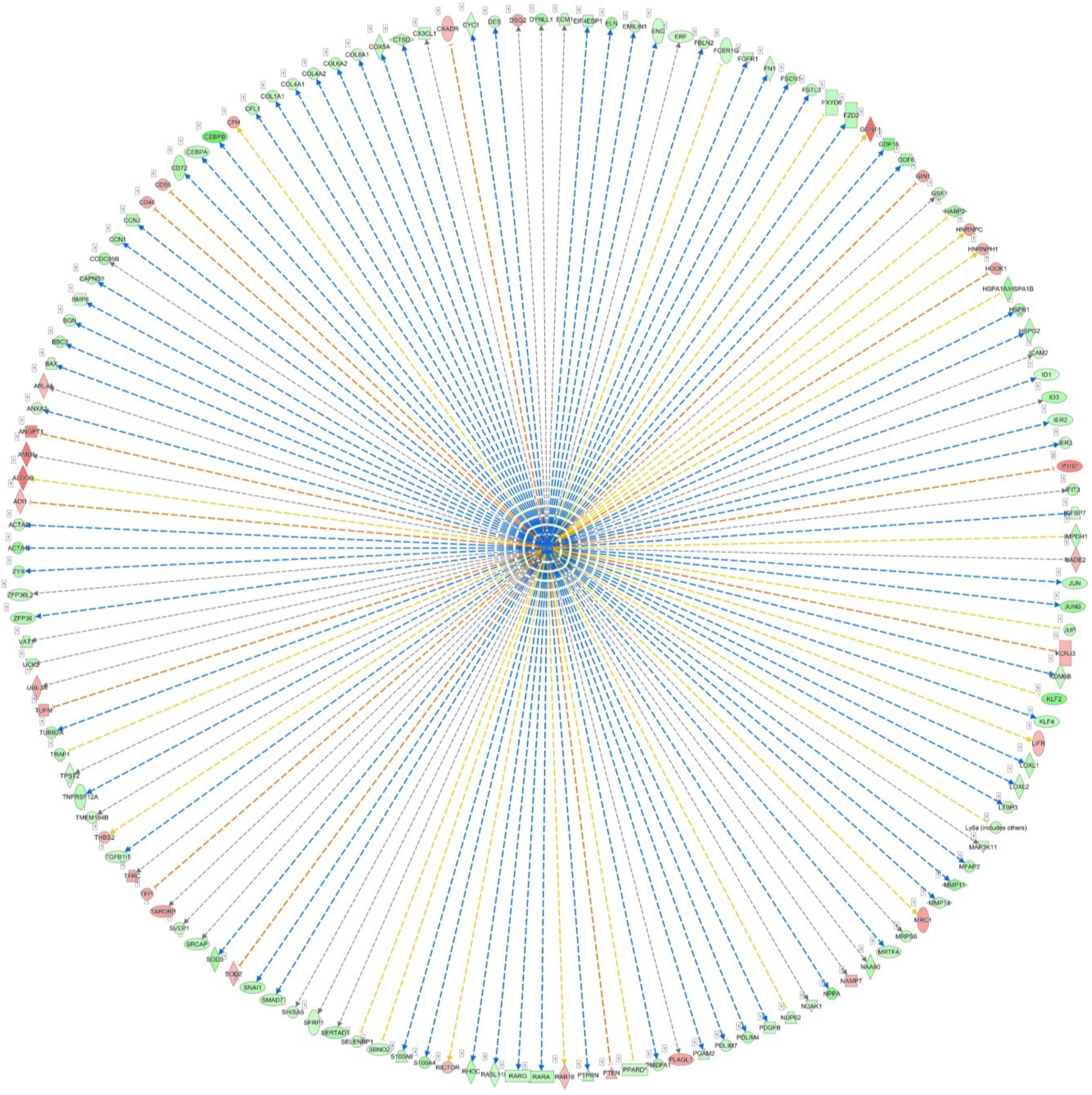

Supplemental Figure 3. TGFB1 is one of the regulator mediates CIP's function and down-regulated in TAC-CIP-OE heart. Ingenuity Pathway Analysis (IPA) of upstream regulators of dys-regulated genes in CIP-OE heart after 8 weeks of TAC operation. Genes in green indicate their expression in CIP-OE heart is down-regulated. Genes in red indicate their expression in CIP-OE heart is up-regulated. Blue lines indicate that the dysregulation of upstream regulator lead to the down-regulation of the downstream genes, which is consistent with reported data; Red lines indicate that the dysregulation of upstream regulator lead to the up-regulation of the downstream genes, which is consistent with reported data. Yellow lines indicate that the gene regulation is inconsistent with reported data. Gray lines indicate the unknown gene regulation.

Supplemental Table 1. Information of controls and dilated cardiomyopathy (DCM) patients

	Gender	Age	EF（Teich）
**Control 1**	Male	35	N/A
**Control 2**	Male	50	N/A
**Control 3**	Female	44	N/A
**DCM 1**	Male	41	30
**DCM 2**	Male	55	20
**DCM 3**	Male	48	51
**DCM 4**	Male	53	29
**DCM 5**	Male	44	15

Supplemental Table 2. Sequence information of qPCR primers used in this study

Species	Gene name	Forward primer (5'→3')	Reverse primer (5'→3')
mouse	NPPA	CACAGATCTGATGGATTTCAAGA	CCTCATCTTCTACCGGCATC
mouse	NPPB	GTCAGTCGTTTGGGCTGTAAC	AGACCCAGGCAGAGTCAGAA
mouse	FBN1	CCTTCCTGTGGCTCCAGAT	GCTGCCCCCATTCATACA
mouse	Acta1	GCCCATCTATGAGGGCTATG	AATCTCACGTTCAGCTGTGG
mouse	Tgfb1	AAGACTTCACCCCAAAGCTG	GAGAGAGGGTCCTGGGATG
mouse	Des	GCGTGACAACCTGATAGACG	TGGATTTCCTCCTGTAGTTTGG
mouse	Bax	GTGAGCGGCTGCTTGTCT	GTGGGGGTCCCGAAGTAG
mouse	Cebpa	CGCTGGTGATCAAACAAGAG	GGTGGCTGGTAGGGGAAG
mouse	Cebpb	TGATGCAATCCGGATCAA	CACGTGTGTTGCGTCAGTC
mouse	Cox6a2	GAGCGCCCAGAGTTCATC	TGTGGAAAAGCGTGTGGTT
mouse	Cox8b	AGCCAAAACTCCCACTTCC	GAACCATGAAGCCAACGAC
mouse	Rictor	GGTGATAACTACGTTCGTCGC	AAAGGTGTACGGGCAGGTAG
mouse	Bbc3	TTCTCCGGAGTGTTCATGC	TACAGCGGAGGGCATCAG
mouse	Ndufa1	TGATGGAACGCGATAGACG	GCCAGGAAAATGCTTCCTTA
mouse	Ndufb9	TTTCCAAGAGAGAGCAGTGGA	CTCCTGCAGCTGCTTAACCT
mouse	Uqcr10	ACTTCCACCTTTGCCCTCAC	TCCACAGTTTCCCCTCGTTG
mouse	Uqcr11	CCACAGGCCTCGATGGTA	GCAGCCCTAGTGTCTGTCAA
mouse	Cox5a	TTAAATGAATTGGGAATCTCCAC	GTCCTTAGGAAGCCCATCG
mouse	Sod3	CTCTTGGGAGAGCCTGACA	GCCAGTAGCAAGCCGTAGAA
mouse	Junb	CCACGGAGGGAGAGAAAATC	AGTTGGCAGCTGTGCGTAA
mouse	Fgfr1	GACCTACGTTCAAGCAGTTGG	TCCAGCGGTATGGACAGG
mouse	Smad7	ACCCCCATCACCTTAGTCG	GAAAATCCATTGGGTATCTGGA
mouse	Rhoc	AAGGACCTGAGGCAAGATGA	AAGGCACTGATCCTGTTTGC
mouse	Pdgfb	CGAGGGAGGAGGAGCCTA	GTCTTGCACTCGGCGATTA
mouse	Igfbp7	TGCCCTCCATGAAATACCAC	GGCTGTCTGAGAGCACCTTT
mouse	CIP	TAGCTACTCGGCCCAAGTCT	ATCCCATGAGGAATTTCAGG
mouse	PRKG1	TCCAACATTCCAGAGCCTTC	TTTTCATAGTGGGTCTCTTCGAG
mouse	β-actin	GATCTGGCACCACACCTTCT	GGGGTGTTGAAGGTCTCAAA
